# 

**Published:** 1888-07-14

**Authors:** 


**The Doctor's Pulpit.**—The Uses of a Health Resort - 239

**Hints for the Sick-room.** By M. M. BASIL, M.A., M.B., Author of "The Commoner Accidents to Life and Limb."—Poultices, Fomentations, Sinapisms, Lotions, etc. - 240

**The Hospitals Association and Patients' Contributions** - 241

**Phlebotomy in Excelsis.** BY DR. W. CURRAN - 242

**Notes and News** - 244

**Everybody's Page** - 246

**Annotations.**—Profane Demonstrations by Friendly Societies.—Nurse Farming - 247

**In and Out Among the Hospitals.** By a SECRETARY OF TEN YEARS' STANDING.—St. George's Hospital.—The Suffolk General Hospital.—Queen's Hospital, Birmingham.—Westminster Hospital - 248

**An Ishmaelite.** By LESLIE KEITH, Author of "The Chilcotes," "East and West," etc. - 249

**The Paddington Fund for the Unemployed** - 251

**Amusements with Profit for all Ages.**—For Men and Women.—Children's Corner.—Puzzles, etc. - 252

**EXTRA SUPPLEMENT.—THE NURSING MIRROR.**

En Passant —Staffordshire Institution for Nurses.—The Women's Jubilee Offering.—Mrs. Ernest Hart.—School of Medicine for Women —The New Hospital for Women - lvii
District Nursing - lviii
St Mary's Infirmary, Notting Hill - lix
Anecdotes of Personal Adventure.—A Mauvais Quart d'Heure.—"Als Well that Ends Well." - lix
Everybody's Opinion.—Patients and Visitors - lx
Appointments - lx
Vacancies - lx

